# Diffuse Destructive Changes in the Liver Associated with Jaundice

**Published:** 1910-03

**Authors:** Carey Coombs

**Affiliations:** Assistant Physician to the Bristol General Hospital.


### DIFFUSE DESTRUCTIVE CHANGES IN THE LIVER ASSOCIATED WITH JAUNDICE.

BY

CAREY COOMBS, M.D., M.R.C.P. Lond.,
*Assistant Physician to the Bristol General Hospital.*

---

THERE is not such a lack of writings on this subject as to justify a mere record of cases seen, especially when several of the cases have already been published; and since the observation of these three cases, which I am about to describe for the first time (in addition to three already on record), only devolved in part upon myself, it is necessary to give a passable excuse for the construction of this paper. My reason is this, that within seven years I have had the very unusual experience of seeing six examples of a not common group of disorders, with the result that certain definite impressions regarding the essential features of these cases remain on my mind, impressions which seem worth setting down in black and white.

**Case 1.**—M.S., female, 25, married, three children, youngest five months; pregnancy, confinement and puerperium natural. The illness began on March 7th, 1906, with weakness and jaundice but no pain. The jaundice deepened, she vomited a few times and had a little pain in the back. On March 21st she came into the General Hospital under Dr. Parker. She was at that time deeply jaundiced, but with a clean tongue, a pulse of 100 and no fever. The edge of the liver was felt two inches below the costal border; it was smooth and not tender. On March 23rd the pulse quickened to 156; she became delirious and died two days later in coma, with a temperature of 107°.

At a *post-mortem* examination next day the tissues were deeply jaundiced, but not wasted or œdematous, and there was no ascites; the liver weighed 39 oz.; the upper part of the right lobe and the left lobe were shrunken. Over these parts the capsule was wrinkled, elsewhere smooth. The shrunken parts were firm and deep reddish-purple, and lacked the usual lobular pattern. This was, however, visible in the other parts of the liver, which were normal in consistence but deep yellow in colour. The larger biliary passages were normal. The mucous membrane of the small bowel, of the duodenum particularly, was reddened, and under the visceral pericardium were a few petechiæ.

The sections which I cut from the liver showed the following points. The red, shrunken parts contained no liver cells, but the connective tissue framework was left, the lobular outlines being very distinctly defined by tracts of reactive change corresponding with the interlobular septa. The reaction here was of two kinds, connective-tissue proliferation and "bile-duct" formation. The "embryonic bile-ducts" were plentifully scattered throughout these zones, though nowhere else, the inflammatory cells, small fibroblasts, lymphocytes, and plasma cells, being similarly limited in their distribution. The yellow areas were made up of lobules normal in shape, but with every cell more or less necrotic, cloudy and swollen, and wanting in a definite nucleus. The same type of change was found throughout the renal epithelium.

**Case 2.**—M.P., female, admitted to the General Hospital October 1st, 1906, also under Dr. Parker. Five months before admission she was jaundiced for two or three days only. Three months after this the jaundice returned with abdominal pain, vomiting, short breath and swelling of the face. The jaundice continued and steadily increased; she went on vomiting, and was admitted with a tense ascites. The jaundice at that time was moderate, and on her chest were some purpuric spots. On October 9th the abdomen was so tight as to call for drainage; and by way of giving a chance for exploration to clear up the diagnosis, a small incision was made. Much bile-stained fluid ran out; and though the liver had a nodular feel (like new growth), there was no clear reason for further operation, so the wound was closed. Next day the pain in the belly was so bad that morphia gr. $\frac{1}{6}$ had to be given. That evening the breathing became slow, the pulse failed and she became comatose, dying early on the morning of October 11th.

The *post-mortem* inspection showed a general œdema with ascites, but no peritonitis, and hæmorrhages under the skin, and visceral pericardium. The liver, weighing $26\frac{1}{2}$ oz., was flabby and its capsule wrinkled. In many places, especially the under part of the quadrate lobe and the left lobe, were bright yellow patches contrasting sharply with the pale reddish-brown of the rest of the liver. These patches when cut into showed a distinct lobular outline not seen in the rest of the liver. The bile passages were normal, the mucosa of the intestine reddened, and the fæces pale. Sections of the yellow areas showed normally-outlined lobules with every cell moribund and cloudy. The rest of the liver was cut up into compartments by bands of proliferative reaction, that is to say, of fibroblasts and "embryonic bile-ducts." These tracts certainly were interlobular trabeculæ; but as the enclosed spaces were distorted and smaller than natural lobules, some shrinkage must have taken place. In the renal tubules there was cloudy swelling, also in the heart wall.

**Case 3.**—C.I., male, aged 4, came to my out-patient room on January 4th, 1909, with jaundice which had lasted about four days. He was first taken ill with diarrhœa, but no vomiting. The liver was just palpable, the motions were pale and the urine dark. I thought he had catarrhal jaundice, and did not take him in. On January 13th he was no better and the jaundice was deeper, so I admitted him under Dr. Symes, whose notes follow By this time the bowels were rather confined, there was occasional vomiting and slight upper abdominal pain. On January 17th he became drowsy, then passed into a screaming delirium. Twelve hours later, having been partly quieted by treatment, he lapsed into a typhoid-like stupor with convulsive twitchings, and died comatose on January 19th, 1909.

The *post-mortem* examination by Dr. Emrys-Roberts (to whom I am further indebted for the microscopical sections presently described) showed sterile fluid in the pericardium, petechiæ in omenta and mesentery, entero-colitis, and venous congestion of the liver with general jaundice. This organ weighed 18 oz. and displayed no shrunken reddish areas as in the foregoing cases. In the sections every lobule showed necrosis of the more central cells, and at the edge was a mild leucocytosis with "bile-duct" formation.

The other three cases were seen by me. As they have already been publicly recorded, my notes will consist of brief abstracts of the records which have already appeared. Cases 4 and 5 were reported by Sir John Broadbent,[1] Case 6 by Dr. Bertram Rogers in this *Journal.*

**Case 4.**—A girl of 17, admitted January 7th, 1902, to St. Mary's Hospital, under Dr. D. B. Lees (whose house-physician I was at the time). She had for a month suffered abdominal pain and progressive jaundice ; the liver was just palpable and tender. After two or three days in hospital she became delirious, and died in coma on January 12th. The liver, weighing 40 oz., showed widespread cellular necrosis, and though no proliferation was noted, a few shrunken, depressed areas were seen with the naked eye.

**Case 5.**—A girl of 22, admitted to St. Mary's Hospital (while I was medical registrar there) September 29th, 1903, also under Dr. Lees. She had been ill nine months, jaundiced five months, and for three months had had increasing ascites with œdema of

[1] *Tr. Path. Soc. Lond.*, 1905, lvi, 145.

[2] *Bristol M.-Chir. J.*, 1906, xxiv, 40.

the ankles following. By October 3rd the belly was very tense, and it had to be opened. Much fluid escaped, but there was nothing except doubtful nodules to be felt in the liver, so the wound was closed. She became delirious, then comatose, and died on October 5th. The liver consisted of two distinct portions, one dull brick-red in colour and homogeneous, the other composed of irregular polygonal areas of bile-stained lobules. In these latter areas the cells were swollen, and fibrous and granular ; the former consisted of a network of fibrous tissue with many embryonic ducts. The renal tubules also showed cloudy swelling.

**Case 6.**—A boy of 4, admitted to the Children's Hospital on June 2nd, 1905, under Dr. Rogers (while I was registrar to the Hospital). Three months before admission there was an illness, possibly associated with jaundice. He was admitted delirious and deeply jaundiced ; the temperature rose to 108°, and he died comatose on June 4th. During life the liver was felt ; *post-mortem* it weighed 17 oz., about normal at that age ; it was dull, red and firm. Microscopically only a few outlines of liver cells remained ; the lobular boundaries were marked by tracts of fibrosis and bile-duct formation.

Now in four of these six cases I was allowed to study the clinical features, and in all of them the pathological data. I believe they exhibit similarity in certain essentials which I will now try to define. To make this clearer a table of the cases is appended.

The course of the disease falls into two stages. The earlier and longer is one of progressive jaundice. In the two cases where this was longest (2 and 5) there was ascites also, and in all the other cases the liver was felt during this period, which varied from sixteen days to nine months. The final phase lasts two or three days. It begins rather abruptly with delirium, which passes into coma, often with hyperpyrexia, and death ensues.

The pathological changes in the liver also fall into two groups. First there are the areas of firm, red tissue exhibiting proliferation of the stroma and formation of " embryonic bile-ducts." These are partly to be looked upon as evidence of reaction to irritation, partly as an attempt at repair ; in either case they commemorate the activity of some substance which brought them into being, while it destroyed the liver cells, a process which occupied some time. Then there are the yellow, still lobulated areas, in which the liver cells are all in a state of recent necrosis ; these cells would, if the patients had lived long enough, have disappeared, as in the other parts of the liver.

The long stage of progressive jaundice corresponds with the gradual, widespread destruction of liver cells, coupled with the formation of new connective tissue and "bile-ducts"; the terminal toxic stage with the final and complete destruction of such liver cells as had till then been spared. Flexner's[1] experiments throw light on the relation between these two phases. He found that in some rabbits the injection of ricin, abrin and dog's serum caused widespread necrosis of the liver cells with early death. In other animals, who lived longer, the interlobular tissues of the liver showed connective tissue proliferation with formation of "bile-ducts." Analogous lesions are found in man after the action of various poisons, and in several fatal cases of jaundice following appendicitis I have known of similar changes. It seems as if in the cases recorded here the patients had been repeatedly attacked by some poison or poisons unknown, by which their liver cells had been destroyed at a rate varying with the dose of poison and the patient's capacity for resistance. The better the resistance the more marked are the proliferative phenomena. Probably the final stage of intense toxæmia represents not only the complete destruction of cells which are of great metabolic importance, but also the total removal of an agency constantly at work preventing the passage of intestinal and other toxins from the portal to the systemic circulation. In some cases it may be that the same poison at once sweeps away the last surviving hepatic cells and passes on triumphantly into free circulation. In others, perhaps, one poison kills the cells and thus makes the way clear for another.

I have throughout avoided the term "acute yellow atrophy," because I consider it an imperfect description of a group of cases which are not always acute, and in which the liver is necrotic, rather than atrophic, and as red as it is yellow.

My thanks are accorded to my colleagues at the Bristol General Hospital for their kindness in allowing me to record cases 1, 2 and 3.

[1] *Brit. M. J.*, 1900, ii, 912.

| Sex. | Age. | Symptoms of First Period. | Duration of First Period. | Symptoms of Second Period. | Duration of Second Period. | Morbid Changes in Liver. |
|---|---|---|---|---|---|---|
| F. | 25 | Jaundice. Weakness. Pain in back (slight). Vomiting (occasional) | 16 days. | Delirium, then Coma. Jaundice deepened. Hyperpyrexia. | 2—3 days. | Left lobe and upper part of right lobe red and shrunken=fibroblastic reaction of bile-ducts, but no hepatic cells. Rest yellow=necrotic lobules. |
| F. | 19 | Jaundice. Dyspnœa. Abdominal pain. Vomiting. Œdema of face. Ascites. | 3—5 months. | Respiratory and Cardiac failure. Coma. | 2—3 days. | Main mass red-brown=fibroblastic reaction of bile-ducts, with contraction of lobular outlines, and no hepatic cells. Yellow areas=necrotic lobules. |
| F. | 17 | Jaundice. Abdominal pain. Liver palpable. | 1 month. | Delirium to Coma. Hyperpyrexia. Jaundice deepened. | 2—3 days. | Some shrunken areas. Rest of liver yellow=necrotic lobules. |
| F. | 22 | Weakness. Jaundice. Œdema of ankles. Ascites. | 5—9 months. | Delirium to Coma. | 2—3 days. | Dull brick-red portions=fibroblastic reaction of bile-ducts, but no hepatic cells. Bile-stained areas of necrosed lobules. |
| M. | 4 | Jaundice. Weakness. | 3 months ? | Delirium to Coma. Jaundice deepened. Hyperpyrexia. | 2—3 days. | Liver dull red=fibroblastic reaction of bile-ducts, with a few necrotic liver cells. Yellow areas not noted. |
| M. | 4 | Diarrhœa, then constipation. Jaundice, Vomiting. Abdominal pain. Liver palpable. | 17 days. | Jaundice deepened. Delirium to Coma. | 2—3 days. | Liver yellow; all lobules showed central necrosis and peripheral repair. Red areas not present. |

